# Establishment of a New Zealand White Rabbit Model for Lethal Toxin (LT) Challenge and Efficacy of Monoclonal Antibody 5E11 in the LT-Challenged Rabbit Model

**DOI:** 10.3390/toxins10070289

**Published:** 2018-07-12

**Authors:** Duanyang Zhang, Weicen Liu, Zhonghua Wen, Bing Li, Shuling Liu, Jianmin Li, Wei Chen

**Affiliations:** Laboratory of Vaccine and Antibody Engineering, Beijing Institute of Biotechnology, 20 Dongdajie Street, Fengtai District, Beijing 100071, China; DevinZhang0415@163.com (D.Z.); joker0104151687@126.com (W.L.); zhonghuawen2014@163.com (Z.W.); lbingdl@163.com (B.L.); lslsjy2203@sina.com (S.L.)

**Keywords:** lethal toxin, rabbit model, monoclonal antibody, efficacy of 5E11

## Abstract

Anthrax caused by *Bacillus anthracis* is a lethal infectious disease, especially when inhaled, and the mortality rate approaches 100% without treatment. The anthrax antitoxin monoclonal antibody (MAb) 5E11 is a humanized antibody that targets the anthrax protective antigen (PA). The efficacy of 5E11 needs proper animal models. However, anthrax spores are extremely dangerous, so experiments must be conducted under Biosafety Level 3 conditions. Considering the critical effects of lethal toxin (LT) on hosts during infection, we report the establishment of a LT-challenged rabbit model, which caused 100% mortality with a dose of 2 mg PA + 1 mg LF, while a 4 mg PA + 2 mg LF challenge could limit death to within three days. Then, we evaluated 5E11 efficacy against LT. A prophylactic study showed that the i.v. administration of 40 mg/kg 5E11 four days before lethal dose LT challenge could lead to 100% survival. In therapeutic studies, the i.v. administration of 40 mg/kg 5E11 10 min after lethal dose LT challenge could provide complete protection. Overall, we developed a new LT-challenged rabbit model, and our results indicate that 5E11 shows potential for the clinical application in anthrax treatment.

## 1. Introduction

*Bacillus anthracis*, a Gram-positive and spore-forming bacterium, is the etiologic agent of anthrax that can cause fatal human disease. Infection can be established through inhalational, cutaneous, gastrointestinal or injection routes, of which pulmonary exposure leads to nearly 100% mortality without treatment [1]. The virulence factors of this pathogen have two primary parts: the poly-d-glutamic acid capsule and anthrax toxins. The polyglutamate capsule prevents phagocytosis of the bacterium [2,3]. The three toxin constituents generated by *B. anthracis* are protective antigen (PA), lethal factor (LF), and edema factor (EF). The two catalytic constituents, LF and EF, are transported into the cytosol through receptor binding of constituent PA in the form of the lethal toxin (LT) complex, which is made up of LF and PA, and the edema toxin (ET) complex, which is made up of EF and PA [4]. ET hinders neutrophil activity in vivo and impacts water homeostasis, causing edema, while LT disrupts many signaling pathways, influences cellular functions, induces cell death and is closely related to shock and death in severe anthrax infection [5,6,7].

Despite decades of research, anthrax continues to be a threat to both humans and animals. Vaccines are available for prophylactic treatment, but vaccines will not be effective for the first 4–6 weeks post-administration [8,9]. Antibiotics cannot clear toxins in spite of their strong ability against the bacterium. In addition, antibiotic-resistant strains can be generated in vitro in the laboratory [10,11]. These shortcomings put the spotlight on the requirement for new methods to treat anthrax infection and anti-toxin drugs that can be administered when toxin accumulation in the bloodstream occurs. The key role of PA in anthrax infection and in pathogenesis makes it a good target for treatment strategies [12].

5E11, a humanized IgG1(κ) monoclonal antibody (MAb), binds to domain II of PA with an affinity of 6.63 nM. Our previous studies have proven that 5E11 showed strong neutralizing activity on the cell and LT-challenged rat model [13]. However, there is a greater need of animal models to evaluate the efficacy of 5E11. Rabbits are considered to be well-characterized anthrax animal models, but previous studies for the evaluation of anti-toxin therapies mostly used spore challenge [14,15,16,17,18,19,20]. Virulent strains are extremely dangerous, so spore challenge must be limited in Biosafety Level 3 (BSL-3) conditions. Considering the critical effects of LT on hosts during infection, we planned to establish an LT-challenged rabbit model to investigate the effects of LT on rabbits and to discuss the similarities and differences between LT challenge and spore challenge.

We herein report that we are the first to establish a new LT-challenged rabbit model. This model was utilized in the assessment of the prophylactic and therapeutic efficacy of 5E11. The results indicated that 5E11 has the potential to be a treatment against anthrax.

## 2. Results

### 2.1. Establishment of the NZW Rabbit Model for LT Challenge

**Figure 1:**
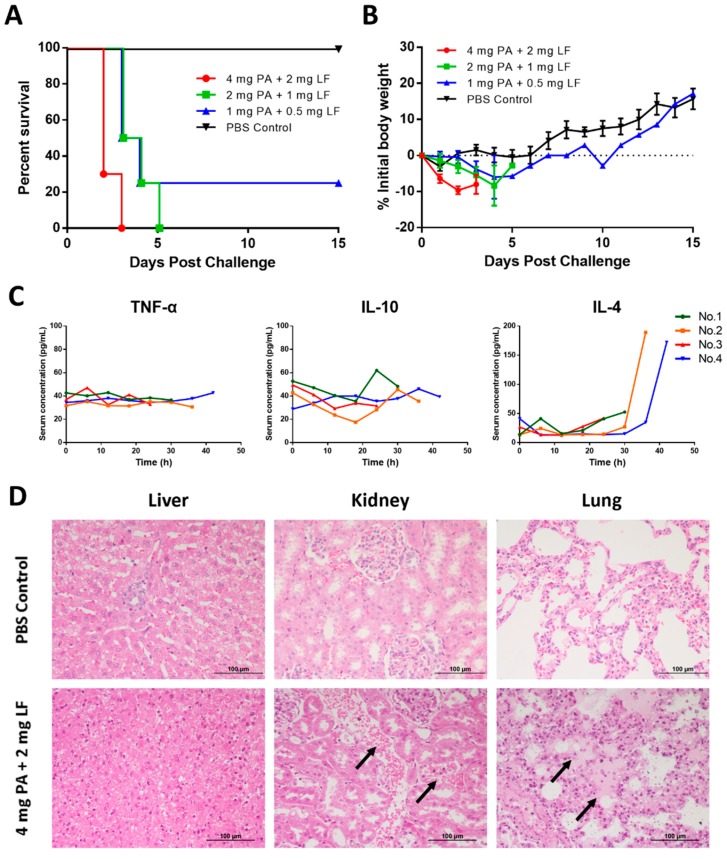
Establishment of lethal toxin (LT)-challenged rabbit model. Rabbits were i.v. injected with 4 mg anthrax protective antigen (PA) + 2 mg lethal factor (LF), 2 mg PA + 1 mg LF, 1 mg PA + 0.5 mg LF, or PBS at day 0. Animals were monitored for 15 days. (**A**) Kaplan-Meier curves representing time to death from challenge and survival data for each group are shown. (**B**) Average weight changes in rabbits. The error bars indicate standard errors of the mean (SEM). (**C**) Blood was taken every 6 h from four rabbits in the 4 mg PA + 2 mg LF group for the detection of TNF-α, IL-10, and IL-4 by ELISA. (**D**) Histological findings. Tissue sections from multiple organs collected from PBS-treated rabbits are all normal. The liver tissue obtained from the rabbits challenged with 4 mg PA + 2 mg LF shows diffuse hepatocellular swelling, narrow liver sinus and necrosis of hepatic cells. In the kidney, arrows show hemorrhaging in renal convoluted tubules in rabbits challenged with 4 mg PA + 2 mg LF. In the lungs, severe edema (arrows) is found in pulmonary alveoli in rabbits challenged with 4 mg PA + 2 mg LF. HE staining, × 20 magnification.

In the LT-challenged rabbit model (LRM), the percentage of rabbits that succumbed to LT increased when challenge dose was higher (Figure 1A). At the highest dose (4 mg PA + 2 mg LF), all the rabbits died between the second and the third day post-challenge, which showed good consistency. In contrast, rabbits that received PBS alone all survived. The 2 mg PA + 1 mg LF group also showed no survival but the average survival time was longer than the 4 mg PA + 2 mg LF group. 2 mg PA + 1 mg LF was approximately confirmed as the minimal lethal dose (MLD). There was one of four rabbits that lived to the terminal timepoint in the 1 mg PA + 0.5 mg LF group.

Clinical signs included declined activity, inappetence, diarrhea, respiratory distress, and unresponsiveness. There were obvious decreases of rabbit body weight in the three LT-challenged groups in the first few days (Figure 1B). Then, the body weight of the only surviving rabbit in the 1 mg PA + 0.5 mg LF group increased in the following days.

The LRM study also provided us data about body temperature (Figure S1) and inflammatory cytokines (Figure 1C). Previously, some reports have demonstrated that body temperature changes in rabbits can be a trigger of therapeutic administration when they are challenged with spores [18,21,22,23]. Here, we did not find out the significant change trends in body temperature in the high dose group (data not shown, Figure S1). We thought that the proliferation and diffusion of the bacteria and the immunogenic capsule made it unique from LT infection, which may potentially clarify the outcomes. To determine whether inflammatory cytokine responses also accompany the LT-induced death and clinical features, serum from high-dose LT-challenged rabbits was analyzed. TNF-α, IFN-γ, IL-1β, IL-4, IL-6, and IL-10 were detected in a pre-experiment, but most of them did not show significant changes. According to the results in the pre-experiment, we chose TNF-α, IL-10, and IL-4 for the experiment. The changes of TNF-α level were not obvious. IL-10 exhibited a rise and fall trend at the final two timepoints before each rabbit’s death. IL-4 showed a significant increase during the last few hours in two of four rabbits. Since IL-4 exhibited both pro- and anti-inflammatory properties, the increase in the late stage was thought to be a signal of inflammatory responses.

Compared to normal findings in PBS-treated animals, tissues obtained from the rabbits challenged with 4 mg PA + 2 mg LF all showed moderate to severe pathological injuries (Figure 1D). In the liver, serious damage, including diffuse hepatocellular swelling, narrow liver sinus, and necrosis of hepatic cells, was observed. Hemorrhaging was found in renal convoluted tubules, and severe edema was found in pulmonary alveoli in rabbits challenged with 4 mg PA + 2 mg LF. Other pathological changes, such as inflammatory cell infiltration could also be observed in tissues.

It is reported that LT can disrupt intestinal barrier function and cause systemic infections with enteric bacteria in mice [24,25,26,27]. Eight rabbits (5 treated with 4 mg PA + 2 mg LF, and 3 with PBS) were included to investigate whether LT could also cause systemic infections with enteric bacteria in rabbits. All PBS-treated rabbits were blood culture negative, while 80% (4/5) moribund rabbits were found blood culture positive: three were positive for *Escherichia coli*, and one was positive for *Pasteurella multocida* (Table S1). We believed that the systemic infections were related to the intestinal barrier injury caused by LT.

### 2.2. 5E11 Pharmacokinetics (PK) in NZW Rabbits

**Figure 2:**
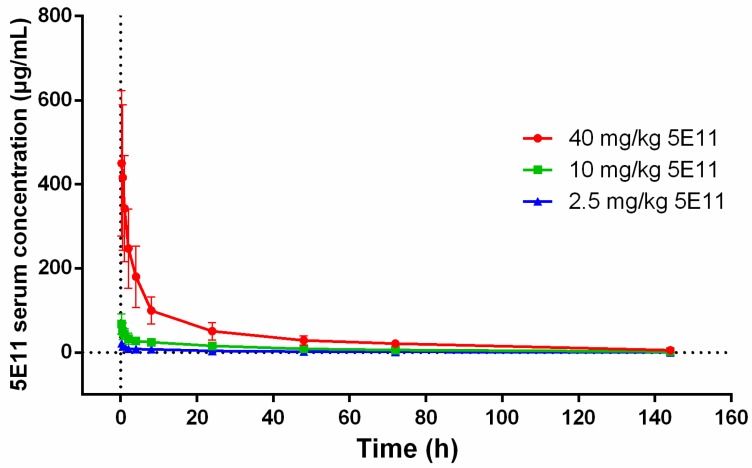
5E11 levels in rabbit serum measured by ELISA. The data are presented as mean 5E11 concentrations with standard errors of the mean (SEM). Three groups of rabbits were i.v. injected with 40 mg/kg, 10 mg/kg, and 2.5 mg/kg 5E11, respectively. Serum samples were collected at 0.25 h, 0.5 h, 1 h, 2 h, 4 h, 8 h, 24 h, 48 h, 72 h, and 144 h post-injection. The pharmacokinetics (PK) profile of 5E11 is not significantly different among the groups. The mean t_1/2_ ranges from 33–40 h.

PK parameters of 5E11 in NZW rabbits after one i.v. administration were assessed (Figure 2, Table 1). A dose-proportional elevation in the serum 5E11 concentration was noted at every dose level. Statistical analysis by analysis of variance (ANOVA) suggested that there was no difference in the elimination half-life (t_1/2_) and total clearance (CL) over the doses ranging from 2.5 mg/kg to 40 mg/kg, which was in accordance with the linear PK. The mean elimination half-life ranged from 33–40 h.

### 2.3. Prophylactic and Therapeutic Efficacy in NZW Rabbits

**Figure 3:**
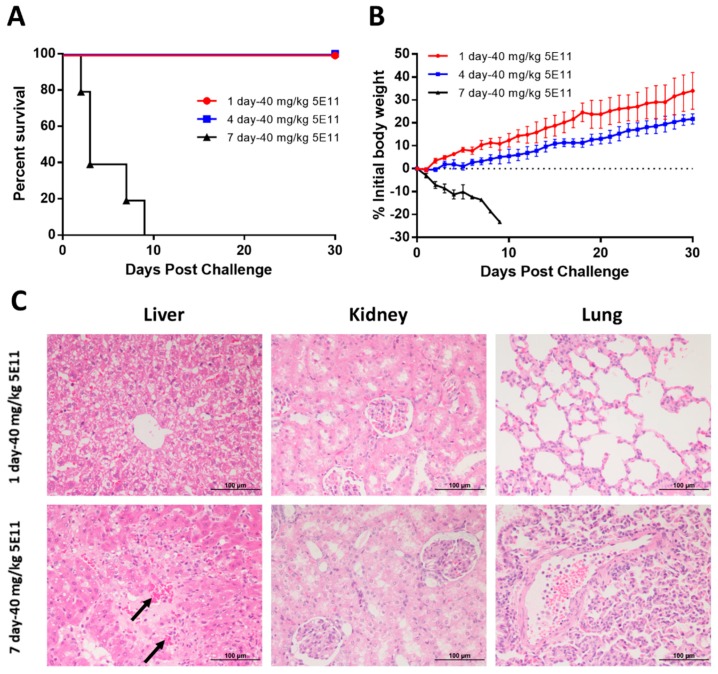
Prophylactic study with 5E11. Rabbits were i.v. administered a single dose of 40 mg/kg 5E11 at 1, 4, or 7 days pre-challenge with 4 mg PA + 2 mg LF. Animals were monitored for 30 days post-challenge. (**A**) Kaplan-Meier curves representing time to death from challenge and survival data for each group are shown. (**B**) Average weight changes in rabbits. The error bars indicate standard errors of the mean (SEM). (**C**) Histological findings. Liver tissues obtained from the rabbits in the one-day 40 mg/kg 5E11 group do not show severe pathological changes. In contrast, the liver tissue obtained from rabbits in the seven-day 40 mg/kg 5E11 group exhibits hemorrhaging (arrows), edema, and large patchy necrosis. The kidney sections of the two groups do not show obvious pathological injury. In the lungs, vasculitis, thickened alveolar walls, and detelectasis of the pulmonary alveoli are identified in rabbits treated with 40 mg/kg 5E11 at seven days pre-challenge. HE staining, × 20 magnification.

5E11 efficacy in the prophylaxis setting was initially examined in rabbits (Figure 3A). The administration of 5E11 at doses of i.v. 40 mg/kg one day and four days before lethal LT challenge (4 mg PA + 2 mg LF) led to 100% survival. The survival rate was 0% (0/5) in rabbits that received 40 mg/kg 5E11 at seven days pre-challenge. Clinical features showed that the body weight of the seven-day pre-exposure group declined rapidly compared with the obvious increase in the one-day and four-day pre-exposure groups (Figure 3B). However, the body weight of the one-day pre-exposure group increased greater than the four-day pre-exposure group. Pathological analysis showed that the damage was more serious in the seven-day 40 mg/kg 5E11 group (Figure 3C). The liver tissue exhibited hemorrhaging, edema, and large patchy necrosis. In addition, vasculitis, thickened alveolar walls, and detelectasis of the pulmonary alveoli were found in the lungs. The results showed that the prophylactic efficacy is better when the prophylactic time is shorter.

**Figure 4:**
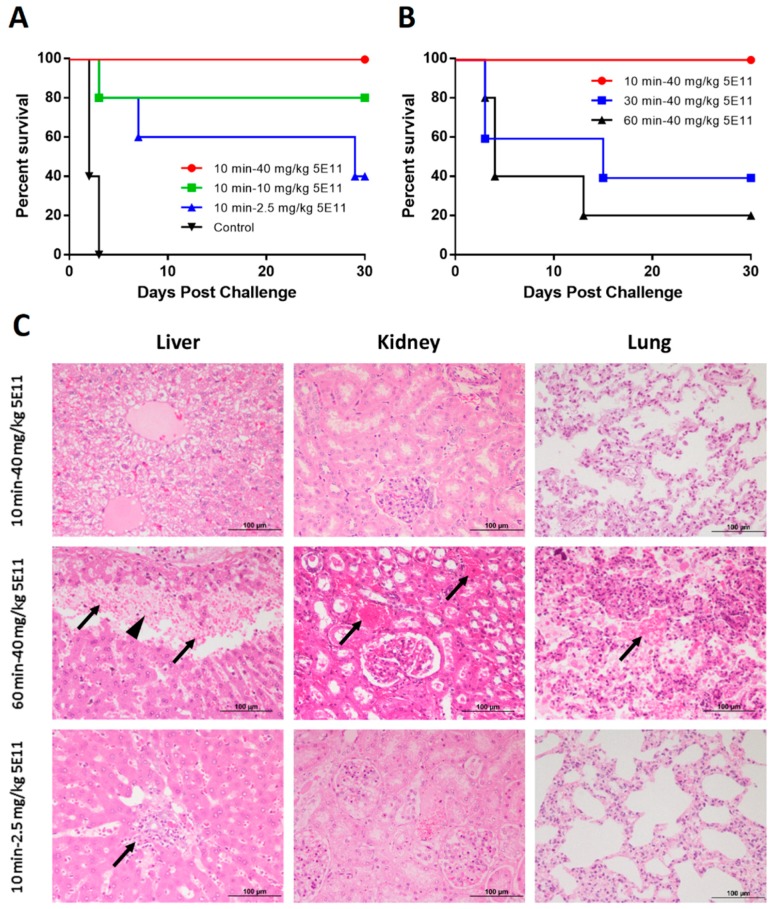
Therapeutic studies with 5E11. (**A**) Rabbits were i.v. administered a single dose of 40 mg/kg, 10 mg/kg, or 2.5 mg/kg 5E11 10 min after being challenged with 4 mg PA + 2 mg LF, and animals were monitored for 30 days post-challenge. Kaplan-Meier curves representing time to death from challenge and survival data for each group are shown. (**B**) Rabbits were i.v. administered a single dose of 40 mg/kg 5E11 at 10 min, 30 min or 60 min after being challenged with 4 mg PA + 2 mg LF, and animals were monitored for 30 days post-challenge. Kaplan-Meier curves representing time to death from challenge and survival data for each group are shown. (**C**) Histological findings. In the liver, mild hepatocellular swelling is found in rabbits in the 10 min-40 mg/kg 5E11 group; hemorrhaging (arrow) and severe necrosis of hepatic cells (arrowhead) are found in rabbits in the 60 min-40 mg/kg 5E11 group; periarthritis in the portal tracts (arrow) is found in rabbits in the 10 min-2.5 mg/kg 5E11 group. In the kidney, hemorrhaging (arrow) is identified in rabbits treated with 40 mg/kg 5E11 60 min post-challenge, while the tissues in the other two groups do not show obvious pathological injury. In the lungs, diffuse hemorrhaging (arrow) and edema are found in rabbits in the 60 min-40 mg/kg 5E11 group. In contrast, no obvious pathological changes are found in the other two groups. HE staining, × 20 magnification.

Two studies, involving Postexposure 1 and Postexposure 2, were conducted to investigate 5E11 efficacy in therapeutic treatment. In Postexposure 1, the i.v. administration of 2.5, 10, and 40 mg/kg 5E11 at 10 min following lethal LT challenge (4 mg PA + 2 mg LF) led to the survival rates of 40% (2/5), 80% (4/5), and 100% (5/5), respectively, compared to 0% (0/5) survival in the Control group (Figure 4A). There were significant differences between the 10 min-40 mg/kg 5E11 group and the Control group (*p* = 0.0023). These results showed a dose-dependent survival rate, and that 5E11 could provide complete protection at a single dose of 40 mg/kg. In Postexposure 2, the i.v. administration of 40 mg/kg 5E11 at 30 min and 60 min post-challenge led to 40% (2/5) and 20% (1/5) survival compared to 100% survival with the administration of 40 mg/kg 5E11 at 10 min post-challenge (Figure 4B). The results showed that the survival was reduced with the delayed administration time. The 10-min therapeutic time window in the LT-challenged rabbit model was very short compared with those in spore-challenged rabbit models in other reports [15,16,17,18,19]. This was one of the different features between LT challenge and anthrax spore challenge. We thought this could be because of the fast cellular uptake of LT (in under 45 min) [28], after which time the antibodies were not able to perform their neutralizing activity due to their feeble capability of passing through the cell membrane.

Pathological analysis also showed that the administration time and dosage could affect the efficacy of 5E11 (Figure 4C). Multiple tissues in rabbits treated with 40 mg/kg 5E11 at 10 min post-challenge did not show obvious pathological changes. In contrast, the pathological changes in rabbits in the 60 min-40 mg/kg 5E11 group included hemorrhaging and severe necrosis of hepatic cells in the liver, hemorrhaging in the kidney, and diffuse hemorrhaging and edema in the lungs. In the 10 min-2.5 mg/kg 5E11 group, periarteritis in the portal tracts was observed while no obvious pathological changes were found in the kidney and lungs. The results indicated that the administration time may be more influential for the efficacy of 5E11 compared to the dosage in LT-challenged rabbit model.

**Figure 5:**
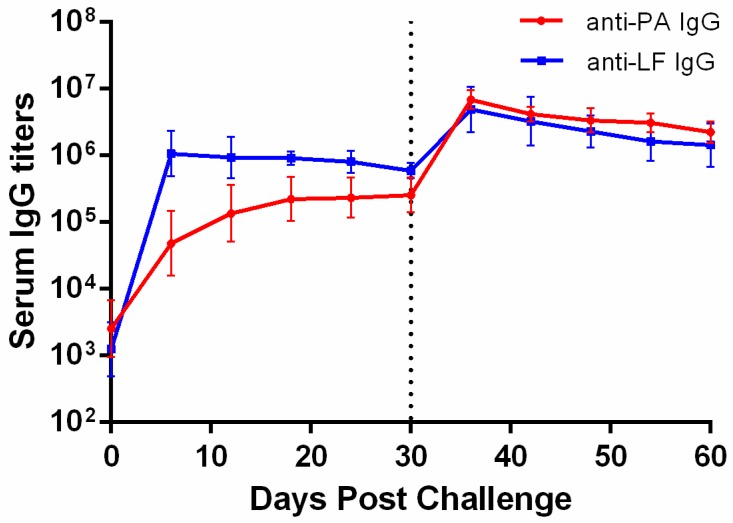
Serum anti-PA and anti-LF IgG titers in rabbits. The data are geometric mean with error bars representing the 95% confidence interval. All of the rabbits in the 10 min-40 mg/kg 5E11 group were rechallenged with 4 mg PA + 2 mg LF for the second time on Day 30 post-challenge and they were monitored for another 30 days. During the entire 60 days, blood was taken from rabbits every six days for the detection of rabbit anti-PA and anti-LF polyclonal IgG by ELISA.

Monoclonal antibody treatment is passive immunity, thus we wanted to know whether rabbits could establish self-immunity responses to lethal LT challenge. The rabbits in the 10 min-40 mg/kg 5E11 group were i.v. injected with 4 mg PA + 2 mg LF for the second time, 30 days following the first challenge. No rabbits died following the rechallenge and there were no obvious clinical signs during the experiment. Blood samples were collected during the entire 60 days for the detection of rabbit anti-PA and anti-LF polyclonal IgG by ELISA. Results are shown in Figure 5. Anti-PA and anti-LF titers increased significantly on Day 6 post-challenge. Anti-LF IgG values were more than 10 times higher than anti-PA IgG values. Anti-PA and anti-LF IgG values did not change much in the subsequent 24 days. After the LT rechallenge on Day 30, anti-PA and anti-LF IgG titers were both increased by approximately one order of magnitude. The results showed that anti-LT self-immunity responses were established in rabbits.

## 3. Discussion

Antibiotics are still used as the main molecules for anthrax treatment, but they have limitations. They cannot clear toxins, which play a critical role in individual death. Vaccines have been proven to be effective in prophylaxis against anthrax, but it takes a long time to establish strong immunity responses after inoculation [8,9]. Antibodies against anthrax are studied widely in recent years, especially after the anthrax attacks in 2001 in the US [4]. There are currently two FDA-approved MAbs (raxibacumab and obiltoxaximab) that are administered along with antibiotics for inhalational anthrax and for the prevention of anthrax when alternative therapies are not accessible or appropriate [18]. 5E11, developed for the treatment of anthrax, neutralized PA strongly on the cells and LT-challenged rat model in previous studies [13]. However, there is still a great need of animal models to evaluate the efficacy of 5E11. In this article, we built a LT-challenged rabbit model and we used it to assess 5E11.

In part of the model establishment, the rabbits were i.v.-challenged with three dose levels of LT. The results showed a dose of 1 mg PA + 0.5 mg LF could be lethal to rabbits. A dose of 2 mg PA + 1 mg LF (1 × MLD) could cause 100% mortality within five days, and the high dose of 4 mg PA + 2 mg LF (2 × MLD) could limit the rabbits’ death time between 24–72 h, which showed good consistency. In addition, we used IPTT-300 to detect the body temperature of some rabbits who received the high dose of LT during the study, but we did not find significant changes as in the rabbits with spore challenge [18,21,22,23]. The differences in body temperature between LT challenge and spore challenge may be due to the proliferation and diffusion of spores and their immunogenic capsule, which could better induce immunity responses [21,22]. Then, inflammatory cytokines were detected, but we did not find significant change trends in TNF-α, IFN-γ, IL-1β, IL-6, or IL-10. The concentrations of IL-4 increased significantly a few hours prior to death in two of four rabbits. Due to the pro- and anti-inflammatory properties of IL-4, the increased level of IL-4 showed a strong inflammatory response in the late stage of infection. Combined with the results in the pathological analysis, we speculated that the inflammatory response may be caused by the severe damage to multiple tissues and organs in the dying rabbits. The pathological analysis here showed that severe damage, including diffuse hepatocellular swelling, narrow liver sinus, and necrosis of hepatic cells, was observed in the livers in rabbits challenged with 4 mg PA + 2 mg LF. In addition, hemorrhaging was found in renal convoluted tubules and severe edema was found in the pulmonary alveoli.

According to previous reports, anthrax toxins in hosts could reach high levels during anthrax spore infection [18,19,29,30]. The blood volume of a rabbit is about 70–100 mL per kilogram, so when a 2-km rabbit was challenged with LT (1 × MLD to 2 × MLD), the highest concentration of PA in the blood could reach 10^4^ ng/mL by calculation. It was comparable to the peak concentration of PA detected in rabbit serum after inhaled Ames spore challenge with a lethal dose, although taking into account the distribution in tissues [29]. This could be a connection between LT challenge and spore challenge in rabbits and indicated that our LT-challenged model may in some way simulate the situations in the late stage of anthrax spore challenge.

In addition, it was found that most LT-challenged rabbits became systemically infected with enteric bacteria prior to death. Similar findings have been reported in LT-challenged mice [24,25,26,27]. We speculated that LT could disrupt intestinal barrier function, causing systemic infections with enteric bacteria, and LT’s damage may be not species-specific.

The efficacy of 5E11 against LT in the rabbit model included one prophylactic study and two therapeutic studies. First, PK parameters of 5E11 in rabbits were analyzed. The mean elimination half-life of 5E11 in rabbits was approximately 1.5 days. The prophylactic study showed that the prophylactic time window of full protection was approximately four days. The administration of 40 mg/kg 5E11 at seven days pre-challenge did not save any rabbits (0/5) and the pathological analysis showed that the liver and lungs were damaged more seriously. However, compared with the 4 mg PA + 2 mg LF group in the LRM study, the average survival time in the seven-day 40 mg/kg 5E11 group was longer.

Then, 5E11 was evaluated in therapeutic studies. In Postexposure 1, the administration of 40 mg/kg 5E11 at 10 min post-challenge could provide 100% protection and the results showed a dose-dependent survival rate. When the dose was reduced to 10 mg/kg and 2.5 mg/kg 5E11, the survival rate was 80% and 40%, respectively. In Postexposure 2, when the dose was 40 mg/kg, the results indicated that the therapeutic time window was approximately 10 min, and it showed a time-dependent survival rate from 10–60 min (30 min and 60 min led to 40% and 20% protection, respectively). It has been documented that the fast cellular uptake of the LT and the subsequent toxic effects induced by LF could take place in under 45 min in vitro [28], but most of the antibodies were not able to pass through the cell membrane or act even if the administration was a little later on. This may explain the short therapeutic time window with LT challenge. It is considered as one of the different features between LT challenge and spore challenge. Later, a re-challenge experiment showed that the survival rabbits could establish self-immunity responses after the first LT challenge. Pathological analysis showed that tissues obtained from the rabbits in the 10 min-40 mg/kg 5E11 group exhibited no severe pathological changes. The tissues were damaged more seriously in the 60 min-40 mg/kg 5E11 group than those in the 10 min-2.5 mg/kg 5E11 group. Combined with the survival rates, the results indicated that the therapeutic time was a more influential factor to the efficacy of 5E11 compared with the dosage in the LT-challenged rabbit model. In fact, the therapeutic time windows of antibodies are critical in the inhalational anthrax infection. Our rabbit model for LT challenge was in some way similar as that with spore challenge in the late infection stage. We believe that antibodies may not have the ability to save hosts’ lives when toxins accumulated in the bodies to some extent. In human cases, anthrax-infected patients go to hospital usually after experiencing flu-like symptoms, which can be several days after the initial infection. Furthermore, it takes time to confirm the anthrax diagnosis and take medical countermeasures. The delayed treatment time may cause increased toxin levels and thus affect the efficacy of antibodies. However, at present, there are not enough human cases to define the therapeutic time window of antibodies, so it has become a crucial subject for researchers [31]. The results in our studies suggest that toxin levels in bodies are closely related to the survival of hosts and should be monitored frequently for the patients infected with inhalational anthrax.

Overall, in this article, we established a new rabbit model for LT challenge and evaluated the model in multiple aspects. The results demonstrated the remarkably high toxicity of LT and its various intense effects on rabbits. The model helped us to evaluate the efficacy of 5E11. One prophylactic study and two therapeutic studies described here indicate that 5E11 neutralizes anthrax toxin efficiently and has the capability of lowering morbidity and mortality related to anthrax.

## 4. Materials and Methods

### 4.1. Study Designs

All of the studies used a randomized and controlled design (Table 2). In the LRM study, rabbits were i.v.-challenged with three dose levels of LT (4 mg PA + 2 mg LF, 2 mg PA + 1 mg LF, and 1 mg PA + 0.5 mg LF) or PBS, which was used for the establishment of the LT-challenged rabbit model. Clinical observation and pathology analysis were included in the LRM study. Four of the ten rabbits that were exposed to the high-dose LT challenge (4 mg PA + 2 mg LF) were involved in the detection of inflammatory cytokines in serum and body temperature during infection. For blood culture, another eight rabbits (five treated with 4 mg PA + 2 mg LF and three with PBS) were included. Blood was collected via auricular artery in moribund rabbits. Next, pharmacokinetics (PK) parameters of 5E11 were obtained. Three groups of rabbits were i.v. administered 5E11 (40 mg/kg, 10 mg/kg and 2.5 mg/kg of body weight, respectively), and serum was collected at various time points for the use of PK analysis.

5E11 efficacy against LT challenge was evaluated in the following three studies. In the Preexposure study, 40 mg/kg 5E11 was i.v. administrated to rabbits at 1, 4, and 7 days, respectively, prior to lethal LT challenge (4 mg PA + 2 mg LF). In post-exposure studies, Postexposure 1 was examined in a dose-ranging study with the administration of 5E11 (40 mg/kg, 10 mg/kg, 2.5 mg/kg, and 0 mg/kg, respectively) in 10 min after lethal LT challenge (4 mg PA + 2 mg LF). In Postexposure 2, 5E11 efficacy was assessed at increasing times post-exposure with the administration of one 40 mg/kg dose in 10, 30, and 60 min after lethal LT challenge (4 mg PA + 2 mg LF). The surviving rabbits who were administered 40 mg/kg 5E11 at 10 min post-challenge were rechallenged with the same lethal dose of LT (4 mg PA + 2 mg LF) to evaluate whether the rabbits could build strong self-immunity responses after the first challenge. ELISA was used to measure the levels of rabbit anti-PA and anti-LF polyclonal IgG. Clinical observation and pathology analysis were also included in the 5E11 efficacy studies. Except for the analysis of PK parameters, the survival rate was the primary endpoint, defined as the proportion of animals alive at the time of scheduled study termination.

### 4.2. Animals

Female New Zealand White (NZW) rabbits, weighing between 1.7 and 2.3 kg, were purchased from Jinmuyang Experimental Animals, Inc. (Beijing, China). Animals were kept in separate cages and were allowed to become accustomed to a 12/12-h light/dark cycle in a temperature- and humidity-regulated area. Food and water were offered *ad libitum*. Each of the rabbits was acclimated to the facility for at least seven days before the challenge or administration. Just the healthy animals within the specified weight range and those that were free of notable clinical signs of disease or malformations were put into the studies. Rabbits were sacrificed at the noted time via CO_2_ inhalation. Each of the experiments was endorsed by the Animal Care and Use Committee of Laboratory Animal Center, Institution of Military Medicine, Academy of Military Science (Approval Number IACUC-DWZX-2017-001).

### 4.3. LT and 5E11 MAb Preparation

Recombinant protective antigen (PA) and lethal factor (LF) were expressed in *Escherichia coli* [32]. After purification, the purity values of PA and LF judged by sodium dodecyl sulfate-polyacrylamide gel electrophoresis analysis were both more than 95%, showing that the PA and LF could be used for the animal experiments and ELISA in our studies. 5E11 was a humanized MAb derived from mice and was expressed by Chinese hamster ovary (CHO) cells. The final purity of 5E11 could reach more than 99%.

### 4.4. LT Challenge and Administration

PA and LF were mixed with a concentration rate of 2:1 prior to use in 1 h. Dosages were confirmed according to the study designs and we made sure they had equal volumes in all of the groups with PBS, including the Control group. Then, the confirmed doses of LT or PBS were injected through the ear veins in different groups. The administration process was similar to that of LT challenge. PBS was added to 5E11 to insure equal volumes in all of the groups, and then the doses were injected through the ear veins.

### 4.5. Clinical Observation

NZW rabbits were observed and their body weight was measured after LT challenge. Rabbits were monitored for clinical signs and changes in activity, respiration, food intake, cacation, alertness, and responsiveness. Body weight was measured once daily. Body temperature was measured in four of 10 rabbits challenged with 4 mg PA + 2 mg LF in the LRM study. Body temperature was recorded via an implantable programmable temperature transponder (IPTT-300; BMDS, Seaford, DE, USA) and a DAS8007 reader (BMDS, Seaford, DE, USA). The temperature was recorded every 6 h, from 0–18 h post-challenge, and every 2 h from approximately 18–72 h post-challenge.

### 4.6. Cytokine Assay

The levels of rabbit cytokines (TNF-α, IL-10, and IL-4) in serum were measured via a sandwich enzyme-linked immunosorbent assay (ELISA) using cytokine-specific antibodies according to the manufacturer’s instructions (Elabscience, Wuhan, Hubei, China). Recombinant cytokines (15.63–1000 pg/mL) represented the standards for calibration, and the detection limit was 9.38 pg/mL.

### 4.7. Pathology

Gross necropsy was conducted on the NZW rabbits that were found dead or that were euthanized throughout the study. Portions of target tissues of several rabbits in every group, including lungs, liver, and kidney, were preserved in 10% neutral buffered formalin until histopathology evaluation. Tissues were cut into approximately 4-μm sections, deparaffinized, rehydrated, stained with hematoxylin and eosin, and viewed by a board-certified veterinary pathologist.

### 4.8. Blood Culture and Bacterial Identification

Blood samples obtained from auricular artery were inoculated into BacT/Alert PF bottles (bioMérieux, Inc., Durham, NC, USA). The bottles were then processed in a BacT/Alert 3D automated blood culture system (bioMérieux, Inc., Durham, NC, USA). The bottles signaled as positive were removed from the instrument, and the samples were inoculated into blood agar plates and MacConkey plates, cultured with 5% CO_2_ at 37 °C for 24 h. Bacterial identifications were performed by the VITEK-2 COMPACT fully automated microbiological system (bioMérieux, Inc., Durham, NC, USA).

### 4.9. Pharmacokinetics (PK) Parameters

Rabbits were injected through the ear veins with different doses of 5E11 in different groups. Blood samples were drawn from rabbits at 0.25 h, 0.5 h, 1 h, 2 h, 4 h, 8 h, 24 h, 48 h, 72 h, and 144 h after 5E11 administration, and the concentration of 5E11 in the serum was defined by a tiered-sandwich ELISA. Briefly, plates were coated with purified PA at 2 ug/mL. Plates were blocked with PBS plus 2% Bovine Serum Albumin (BSA) for 2 h. Dilutions of serum (or a standard curve of 5E11) were placed into the wells and incubated at 37 °C for 1 h. Plates were rinsed with PBS and then incubated with goat anti-human IgG HRP conjugate (Abcam, Cambridge, UK). After 1 h at 37 °C, the plates were rinsed, and peroxidase activity was established by adding HRP substrate, and the optical density was read at 450 nm. Serum concentration values of 5E11 versus time for each animal were analyzed using Phoenix WinNonlin 6.4.

### 4.10. Detection of Rabbit Anti-PA and Anti-LF Polyclonal IgG

The method of detecting rabbit anti-PA and anti-LF polyclonal IgG was similar to that of PK parameters, which is a tiered-sandwich ELISA. Briefly, plates were coated with purified PA or LF at 2 μg/mL. Plates were blocked with PBS plus 2% BSA for 2 h. A series of dilutions of serum was placed into to the wells and incubated at 37 °C for 1 h. Plates were rinsed with PBS and then incubated with goat anti-rabbit IgG HRP conjugate. After 1 h at 37 °C, plates were rinsed, and peroxidase activity was established by adding HRP substrate, and the optical density was read at 450 nm.

### 4.11. Statistical Analysis

Statistical analyses were performed using GraphPad Prism 6. For survival analysis, the Kaplan-Meier curves were plotted, and the log-rank test was used. For the elimination half-life (t_1/2_) and clearance (CL) of 5E11, an analysis of variance (ANOVA) model was performed to determine the differences between groups. A *p* value of <0.05 was considered statistically significant.

## Figures and Tables

**Table 1:** Summary of PK parameters in rabbits.

Parameter ^a^	Results (Mean [SD]) by 5E11 Dose (mg/kg) Group:		
	40	10	2.5
C_0_ (μg/mL)	487 (299)	93 (54)	34 (21)
AUC_0–144 h_ (h·μg/mL)	5268 (3613)	1311 (361)	329 (78)
AUC_0–∞_ (h·μg/mL)	5537 (3541)	1375 (351)	348 (80)
t_1/2_ (h)	34 (7)	33 (3)	40 (4)
MRT (h)	26 (9)	37 (3)	33 (1)
CL (mL/h/kg)	11.1 (9.5)	7.6 (1.7)	7.5 (1.9)
Vz (mL/kg)	583 (541)	369 (112)	436 (158)

^a^ C_0_, 5E11 serum concentration at 0 h; AUC_0–144 h_, AUC from 0–144 h; AUC_0–∞_, AUC from time zero to infinite time; t_1/2_, elimination half-life; MRT, mean residence time; CL, clearance; Vz, apparent volume of distribution during terminal phase.

**Table 2:** Study overview.

Study	Study Design	Treatment Regimen and/or Dose	LT-Challenged Dose	No. of Animals
LRM	LT-challenged rabbit model; dose-ranging study of LT administered via i.v. in challenged animals	N	4 mg PA + 2 mg LF	10
		N	2 mg PA + 1 mg LF	4
		N	1 mg PA + 0.5 mg LF	4
		N	PBS	4
Preexposure	Prophylaxis; i.v. dose administered at 1, 4, and 7 d pre-exposure	40 mg/kg (1 d)	4 mg PA + 2 mg LF	5
		40 mg/kg (4 d)	4 mg PA + 2 mg LF	5
		40 mg/kg (7 d)	4 mg PA + 2 mg LF	5
Postexposure 1	Therapy; i.v. dose-ranging study in challenged animals; dose administered at 10 min post-exposure	40 mg/kg	4 mg PA + 2 mg LF	5
		10 mg/kg	4 mg PA + 2 mg LF	5
		2.5 mg/kg	4 mg PA + 2 mg LF	5
		Control	4 mg PA + 2 mg LF	5
Postexposure 2	Therapy; efficacy of 5E11 administered via i.v. at increasing times post-exposure (10–60 min)	40 mg/kg (10 min)	4 mg PA + 2 mg LF	5
		40 mg/kg (30 min)	4 mg PA + 2 mg LF	5
		40 mg/kg (60 min)	4 mg PA + 2 mg LF	5

(N: none).

## Supplementary Materials

The following are available online at http://www.mdpi.com/2072-6651/10/7/289/s1, Figure S1: Body temperature following challenge with 4 mg PA + 2 mg LF in rabbits, Table S1: Bacterial Blood Culture Results in LT-challenged rabbits.
