# Supplementary material for: Establishment of a New Zealand White Rabbit Model for Lethal Toxin (LT) Challenge and Efficacy of Monoclonal Antibody 5E11 in the LT-Challenged Rabbit Model
Source: Toxins (Basel). 2018 Jul 12;10(7):289. doi: 10.3390/toxins10070289 (PMC6071005; doi:10.3390/toxins10070289)
Supplement: Supplementary file 1 [file toxins-10-00289-s001.pdf]

Supplementary Materials: Establishment of a New Zealand White Rabbit Model for Lethal Toxin (LT) Challenge and Efficacy of Monoclonal Antibody 5E11 in the LT-challenged Rabbit Model

Duanyang Zhang, Weicen Liu, Zhonghua Wen, Bing Li, Shuling Liu, Jianmin Li, and Wei Chen

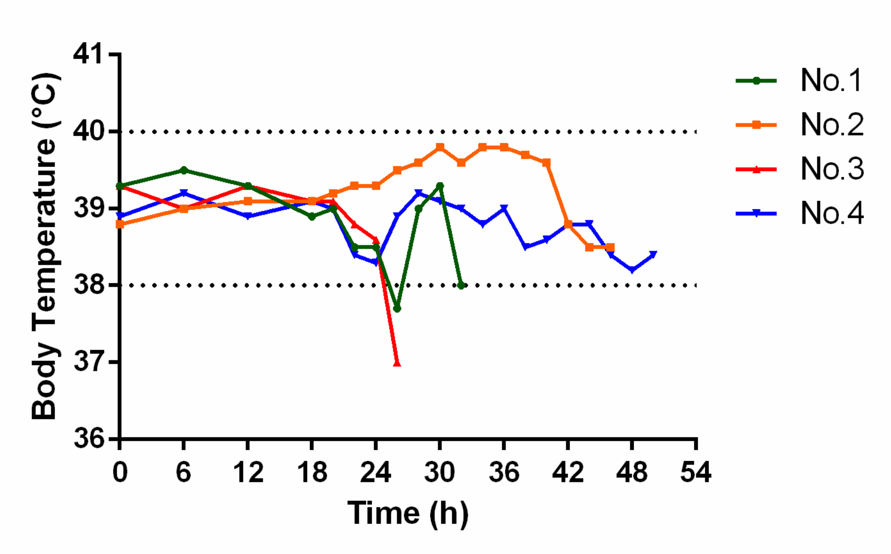

Figure S1. Body temperature following challenge with 4 mg PA + 2 mg LF in rabbits. The body temperature in 4 of 10 rabbits in the 4 mg PA + 2 mg LF group was measured every 6 h from 0-18 h post-challenge, and every 2 h from 18-72 h post-challenge. IPTT-300 and DAS8007 were used to detect the body temperature.

Table S1. Bacterial Blood Culture Results in LT-challenged rabbits*.

Species	N
Escherichia coli	3
Pasteurella multocida	1

*Moribund, LT-challenged rabbits; n = 5 total.
